# Bacteriological quality of effluent submitted consecutively to a macrofiltration and ultraviolet light systems in the Tunisian conditions

**DOI:** 10.1186/s40201-015-0154-6

**Published:** 2015-01-25

**Authors:** Brahmi Mounaouer, Hassen Abdennaceur

**Affiliations:** Water Research and Technology Center, University Tunis Cartage, Borj Cédria Science and Technology Park, P.O. Box 273, Soliman, 8020 Tunisia

**Keywords:** Wastewater, Macrofiltration systems, UV reactor, Disinfection, Reuse

## Abstract

This paper deals with the study of bacteriological quality of effluents that have undergone consecutively different macrofiltration system (pressure sand filter or disc filter used as a secondary treatment) and UV254 irradiation process (used as a tertiary treatment). These two successive systems of treatment were evaluated to determine their possible application as commonly alternatives to the conventional system of wastewater treatment and disinfection before wastewater reuse. They both combined systems of wastewater treatment released effluent of excellent bacteriological quality, with almost total absence of feacal coliforms, of *E. coli* and of *P. aeruginosa*). However, if the bacteriological quality of the effluent remained constant in the case of macrofiltration system (disc filter or pressure sand filter); the UV disinfection process showed to deeply depend on the quality of effluent, particularly with regard to UV transmittance. The daily bacteriological monitoring of the secondary effluent at the exit of the pressure sand filter by UV reactor and by using a dose of 96 mJ/cm^2^, corresponding to an exposure of 16 min, showed an average rate of inactivation of around 3 U-Log, for feacal coliforms, *E. coli* and *P. aeruginosa*, respectively. Therefore, the average bacterial concentration remaining in the water at the exit of the UV reactor is less than 1000 cfu/100 ml for feacal coliform and *E. coli*. For *P. aeruginosa*, the remaining number is less than 100 bacteria/100 ml. These two last values coincide substantially with the range recommended by several standardized international guidelines. Therefore, numerous authors reported that *P. aeruginosa* is very resistant to UV irradiation compared to the other bacterial indicators. In contrast, our study revealed that feacal coliforms and *E. coli* were more UV light resistant than *P. aeruginosa*. This finding could be explained by the fact that *E. coli* and feacal coliform forms aggregates in the treated effluent, while *P. aeruginosa* exists either as discrete cells or as cell pairs.

## Introduction

Reclaim and reuse of urban wastewater have increased in recent years, largely due to lack of water resources and inadequate economic structures, particularly in arid and semi-arid countries [1]. However, the contamination of urban wastewater with pathogenic microorganisms represents a significant risk to public health due to the possible presence of human enteric pathogens. Poor water quality can cause diseases such as gastroenteritis (characterized by vomiting, diarrhea and abdominal pain or fever) or upper respiratory (ear, nose, and throat) infections to exposed swimmers. Highly polluted water can occasionally cause serious diseases such as typhoid fever, dysentery, hepatitis, and cholera [2]. In this sense, to reduce the inconvenience, reuse must be safe to avoid damaging public health and the environment [1].

The usage of treated effluent is considered as an important alternative water resource. Although conventional treatment processes, i.e. primary treatment, disc filter and pressure sand filter (macrofiltration systems) well-known as secondary treatment and recognized to remove up to 99% of microorganisms, were not sufficient to achieve requirements for wastewater discharge and wastewater reuse [3]. The disinfection treatment is considered as the primary mechanism for the inactivation or destruction of pathogenic organisms, to prevent the spread of waterborne disease to downstream users and the environment. Nevertheless, the UV water disinfection efficiency depends on several factors: mainly an adequate contact time and UV intensity to guarantee a sufficient microbial pathogen lessening, In fact, the UV dose is defined as the product of intensity and exposure time [4].

 To date, chlorination is the most widely employed ways to inactivate pathogenic microorganisms in waster and it is considered as the primary process for preventing waterborne infectious diseases throughout the world [1]. However, several studies have reported that the effectiveness of the process is reduced by the water turbidity conditioned by suspended solids and nitrogen compounds such as ammonia and nitrite concentrations [3]. Furthermore, the use of chlorine in wastewater disinfection allowed rising the rate of undesirable and hazardous by-products in both to humans and the environment [5].

On the other side and at the present, UV irradiation is considered as one of the best alternatives to water, chemical disinfection, especially the water chlorination [6]. UV radiations act by interacting damagingly with nucleic acids and other vital cellular components, such as proteins and lipids [7]. The knowledge gained in this field demonstrates that the use of UV for disinfection is a fast, efficient, safe and cost-effective process [6]. The UV irradiation process has been practiced for many years in several countries to disinfect the water [7]. However, microbes have evolved repair mechanisms and can reactivate, once their DNA is partially denatured. Because of their broad wavelength spectrum, the UV lamps, only low or medium pressure, are capable of destroying cellular components such as proteins and enzymes, and so avoiding this reactivation. This fact is justified when the water to be treated must fulfil certain conditions to obtain an optimal effect of UV irradiation. This high failure can be avoided and solved by the application at the beginning of the process, a filtration system such as disc filter or pressure sand filter that assured a good lessening of the main physicochemical parameters such as water turbidity, hardness, suspended solids, iron, manganese, humic acids. All these last parameters were well known as important disruptive factors of UV disinfection [8].

In view of these considerations, the objective of the present work was to evaluate the bacteriological quality (based on the parameters E. *coli,* feacal coliforms and *Pseudomonas aeruginosa*) of different effluents from two macrofiltration processes (pressure sand filter and disc filter) were signaled by lots of authors as pre-treatment. In contrast, in our study, these systems worn as secondary treatment and ultraviolet technologies used as tertiary treatment, in order to ameliorate the physico-chemical and bacteriological characteristics of wastewater disinfection. On the other hand, the aim of this report was to determine the UV irradiation dosages required to destroy E. *coli,* feacal coliforms and *Pseudomonas aeruginosa* in a secondary effluent of a wastewater treatment plant (WWTP), for irrigation purposes.

## Materials and methods

###  Scheme of the experimental system

**Figure 1:**
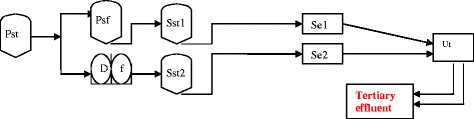
**Pst: Primary settling tank (Influent); Psf: Pressure sand filter; Df: Disc filter; Sst1 and Sst2: Secondary settling tanks; Se1 and Se2: Secondary effluent; Ut: UV treatment.**

Two forms of treatment were set in operation. The first form, constituted with a primary settling and followed by the two macrofiltration processes (pressure sand filter and disc filter), was established in parallel as shown in Figure 1. The second form consisted to a UV treatment process organized as an UV254 monolamp reactor. The pressure sand filter (i) was filled with silica sand of 1 and 3 mm in diameter, and an effective size of 0.8 mm and of high uniformity (*Cu* = 1.6). The filtration system operated with an upward flow at 5.0 m^3^/m^2^ h of hydraulic loading. Cleaning phase was manual, according to load loss (maximum 10 m) and using filtrated water mixed with 10 mg/l of chlorine. Disc filter (ii) was the Arkal battery 3 SKS 2" with an average pore size of 22 μm and an effective surface of 0.282 m^2^, operating at a flow of 4 m^3^/h. The system was equipped with an automatic cleaning system according to load loss (maximum 20 m), and using filtrated water. A cleaning phase using chlorinated (20 mg/l) filtrated water was carried out daily. Feeding of each process was carried out through independent pumping, using water from secondary settling effluent with a range of temperatures between 25 and 30°C.

###  Experimental methodology and analytical determinations

All the systems worked continuously, and samples of water were held daily. Feacal coliforms, *E. coli*, and *P. aeruginosa* were analyzed as bacteriological parameters in all water samples. For bacteriological analysis, water samples were collected in sterile glass bottles of 1 liter and often analyzed within the 48 h after sampling. The presence of thermo tolerant coliforms (feacal coliforms) and *E. coli* was studied using the membrane filtration procedure UNE-EN ISO 9308–1 [9]. Samples (100 ml or dilution) were filtered through Millipore sterile membrane filters (0.45 μm) placed in Petri dishes containing a double layer of tryptic-bile-agar and tryptic-soy agar (Difco), respectively. Petri dishes were incubated at 37 ± 0.5°C for 4 h and then at 44.5 ± 0.5°C for 18 h. The colony count was calculated from the arithmetic mean of three membrane filter counts.

All feacal coliforms isolated in the samples were taxonomically identified, using the bacterial identification system API 20 E (Bio Meriwux, Marcy d’Etoile, France).

Although conventional wastewater treatments are known to remove up to 90–99% of some microorganisms, they may not be sufficiently rigorous and reused effluents may still contain high concentrations of pathogenic microorganisms.

A most-probable-number (MPN) technique was evaluated to detect and enumerate *Pseudomonas aeruginosa* as opportunist pathogen in water and wastewater. Asparagine and acetamid broths were employed as presumptive and confirmatory media in MPN tests, as described in the 13th edition of *Standard Methods for the Examination of Water and Wastewater* [10].

### Sample collection and UV light treatments

Water samples from the secondary effluent at the exit of the disc filter or pressure sand filter were collected in sterile glass bottles for bacteriological analyses. The UV radiation was applied using a low-pressure UVC lamp of 7 Watts (400 mm length and 85 mm diameter), with a flow-rate from 500 to 1100 l/h, and a wavelength of 254 nm. The UV irradiation system was designed to be used with 1000 ml of volume sample and irradiation exposure times of 4, 8, 12, and 16 minutes. According to these conditions, the UV doses provided by the system were of 24, 48, 72, and 96 mJ/cm^2^, respectively. In addition, among the objective of this study was to apply later the selected UV dose for each strain detected in water at the exit of the UV system in order to treat and compare their rates and kinetics of inactivation.

## Results and discussions

### Bacteriological determinations

**Figure 2:**
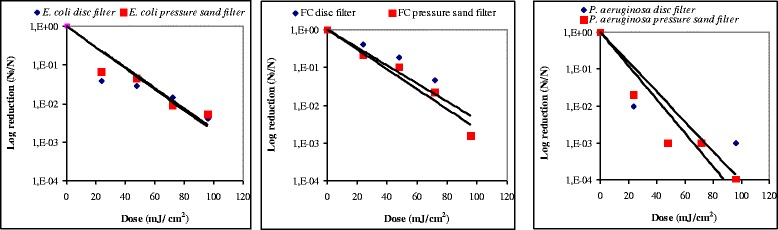
**Comparison of UV inactivation rates for** ***E. coli*** **, feacal coliform and** ***P. aeruginosa*** **(log reduction) at the exit of the disc filter and of the pressure sand filter, respectively.** y: Reduction = *N/N*_*0*_ with *N*; Number of micro-organisms at the instant *T*; *N*_*0*_; Number of micro-organisms at the instant *T* = 0; Dose (mJ/cm^2^) = X = *It* = UV Intensity (mW. cm^−2^). Time of contact(s).

UV radiation is the most commonly used alternative to chlorination, with thousands of installations throughout the world, containing open channels equipped with low or medium pressure mercury discharge lamps. The success of this system can be attributed to high disinfection efficiency for viruses and bacteria, a minimum of disinfection by-products and low cost [11]. As previously indicated, the efficacy of UV disinfection of freshwater and wastewater depends on the UV dose. Because the biological UV dose–response data are generally log-normally distributed, these data are log-transformed [12]. Figure 2 showed the UV inactivation rates for *E. coli*, feacal coliforms and *P. aeruginosa* (log reduction) obtained, respectively, at the exit of the disc filter or pressure sand filter and at different dose UV. From these inactivation curves, the wide divergence noted for *E. coli* could be explained by the poor quality of the influent or the clogging phenomena occurred in the pressure sand filter. For the other two types of bacteria, feacal coliforms and *P. aeruginosa,* we did not remarked any meaningful differences. For this reason, it appeared useful to adopt the secondary effluent at the exit of the pressure sand filter as a model water sample during the bacteriological investigations of the three-selected case of bacteria, and its use in the daily bacteriological monitoring.

**Figure 3:**
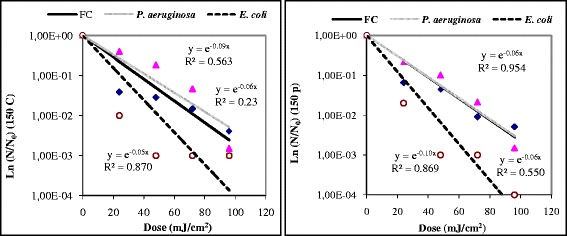
**UV inactivation rates for** ***E. coli*** **, feacal coliform and** ***P. aeruginosa*** **(log reduction).** y: Reduction = *N/N*_*0*_ with *N*; Number of micro-organisms at the instant *T*; *N*_*0*_; Number of micro-organisms at the instant *T* = 0; Dose (mJ/cm^2^) = X = *It* = UV Intensity (mW. cm^−2^). Time of contact(s).

Figure 3 showed the log reduction tendency for feacal coliforms, *P. aeruginosa* and *E. coli* obtained, respectively, at the exit of the disc filter or of the pressure sand filter and at different dose UV. It could be observed that *P. aeruginosa* was more UV resistant than feacal coliforms and *E. coli*, because a higher log reduction of around 4 and 3 U-Log for *E. coli* and feacal coliforms, respectively occurred after an exposure of 16 minutes that corresponds to a dose of 96 mJ.cm^−2^.

All inactivation processes showed a linear portion whatever the dose, despite the lowest correlation coefficient determined for some adjustments. In this sense, to improve some of these adjustments, a linear regression was calculated only with data obtained at lower UV doses (<48 mJ/cm^2^). The correlation coefficient slightly improved for *E. coli*, from 0.87 up to 0.92, for feacal coliforms, from 0.55 up to 0.58, and for *P. aeruginosa*, from 0.97 up to 0.98, leading to a better fitting (results not shown). Authors [13] have identified a gradual flattening at higher UV doses, often attributed to existence of particulate solid matter in the effluent. This point is consistent with the need to apply UV radiations to wastewater previously clarified, in order to avoid the undesirable effect of solid particles [1].

**Figure 4:**
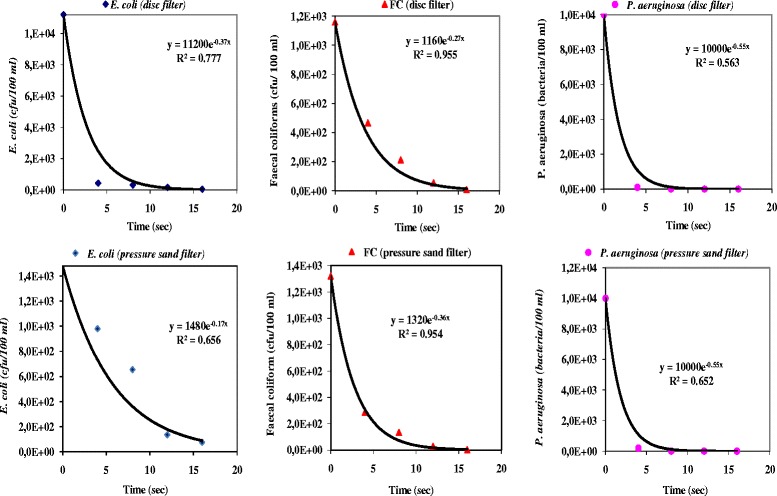
**UV inactivation rates for** ***E. coli*** **, feacal coliform and** ***P. aeruginosa*** **obtained on the exit of the disc filter or pressure sand filter, respectively, and at exposure different times UV.**

Figure 4 showed the UV inactivation rates for the three types of bacteria studied, at the exit of the coupled composed systems, disc filter and UV irradiation or pressure sand filter and UV irradiation, respectively, and by using changed UV doses. The percentage of removal, for the three types of bacteria, increased as the UV time exposure increased. In this sense, effluent samples irradiated during 4 min finalized a reduction of *E. coli,* of feacal coliforms and of *P. aeruginosa,* of around of 97, 94 and 78%, respectively. The removal percentages slightly improved for *E. coli*, from 94 up to 96%, for feacal coliforms, from 78 up to 80%, and for *P. aeruginosa*, from 97 up to 99%, and during 8 min of UV exposure. After 12 or 16 min, almost all of *E. coli,* feacal coliforms and *P. aeruginosa* appeared totally inactivated with any colonies on the growth media. The effect of UV dose on bacteria inactivation is shown in Figure 4. Data fitted to an exponential equation, with correlation coefficients of (0.78; 0.89) for *E. coli*, (0.96; 0.96) for feacal coliforms and (0.57; 0.66) for *P. aeruginosa,* for water samples taken at the exit of the filter disc and pressure San filter, respectively.

**Figure 5:**
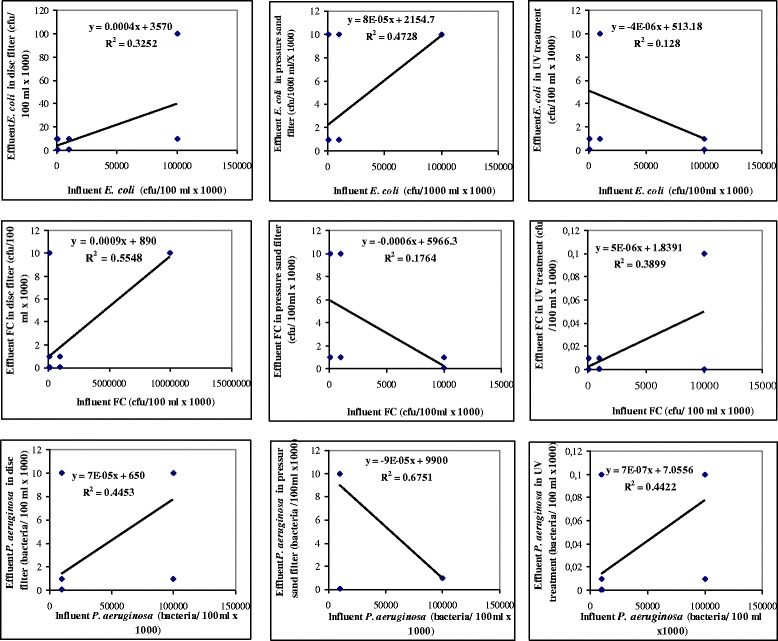
**Effluent concentrations of** ***E. coli,*** **feacal coliform (FC) and** ***P. aeruginosa*** **in disc filter and pressure sand filter as a function of influent concentration of** ***E. coli,*** **feacal coliform (FC) and** ***P. aeruginosa*** **(Linear adjustment).**

Figure 5 showed the inactivation rate of *E. coli,* of feacal coliform and of *P. aeruginosa* for effluent concentration for the disc filter, pressure sand filter and UV reactor, as a function of the *E. coli,* feacal coliform and *P. aeruginosa* concentration in the influent, as linear adjustment. Except for minor cases, a more or less acceptable correlation coefficient was observed in the other cases, showing that concentration of *E. coli,* feacal coliform and *P. aeruginosa* in the effluent is highly dependent with the bacterial concentration of influent. Similar slopes are observed for the majority of cases, with the macrofiltration systems (disc filter and pressure sand filter) presenting similar removal performance for *E. coli*, feacal coliform and *P. aeruginosa*. If the disc filter and the pressure sand filter presented an average retention of *E. coli,* feacal coliform and *P. aeruginosa*, with results of (64 and 62%), (50 and 50%) and (62.5 and 62.5%), respectively; UV represented high elimination percentages for *E. coli* with 87%, for feacal coliforms with 84% and *P. aeruginosa* with 87.5%, for 8 min UV exposure. After 12 or 16 min of UV exposure, *E. coli,* feacal coliforms and *P. aeruginosa* appeared almost all inactivated since any colonies looked in the agar growth media.

This ultraviolet dose is much higher than that reported by Gómez *et al.* [1], who indicated a value over 35 mJ/cm^2^ for total removal (*N/N*_*0*_ = 0) of feacal coliforms, of *E. coli* and of coliphages from municipal wastewater. These authors used a physicochemical coagulation-flocculation water clarification before UV disinfection, allowing an excellent effluents quality with a water turbidity of around 99%. In this study and basing on the Figure 3, a dose of 35 mJ/cm^2^ would remove on average around 37.23 up 50% of *E. coli*, 27 up 37.5% of feacal coliform and 10 up 17.5% of *P. aeruginosa*. These results are also consistent with those reported by Andreakis *et al.* [13] who found a significant increase in the UV disinfection efficiency in sand-filtered water samples. The UV disinfection of water always required secondary effluents of the large degree of clarity by reducing suspended solids content and turbidity. Sharrer *et al.* [14] reported a complete inactivation of coliform bacteria at approximately 77 mJ/cm^2^, in a recirculating salmonid culture system. However, these authors indicated that for total heterotrophic bacteria lessening in the recirculating system required in excess a UV dosage of 1800 mJ/cm^2^ to achieve a quite 2 Log10 reduction. In our experiments, UV doses have been always less than 100 mJ/cm^2^. According to Figure 4, a reduction down to 1000 cfu/100 ml for *E. coli and* feacal coliform and 1000 bacteria/100 ml for *P. aeruginosa* would need a dose over than 24 mJ/cm^2^. An almost total elimination of these indicator bacteria from water would be achieved with doses over than 96 mJ/cm^2^.

### Bacteriological validation of Macrofiltration systems

Conventional wastewater treatments are traditionally known to remove up to 90–99% of microbes that may not be sufficiently rigorous for safe wastewater quality and for agronomic reuse. In this study, we preconized and considered useful a daily bacteriological monitoring of three customary microbes, namely feacal coliform, *E. coli* and *P. aeruginosa*.

**Figure 6:**
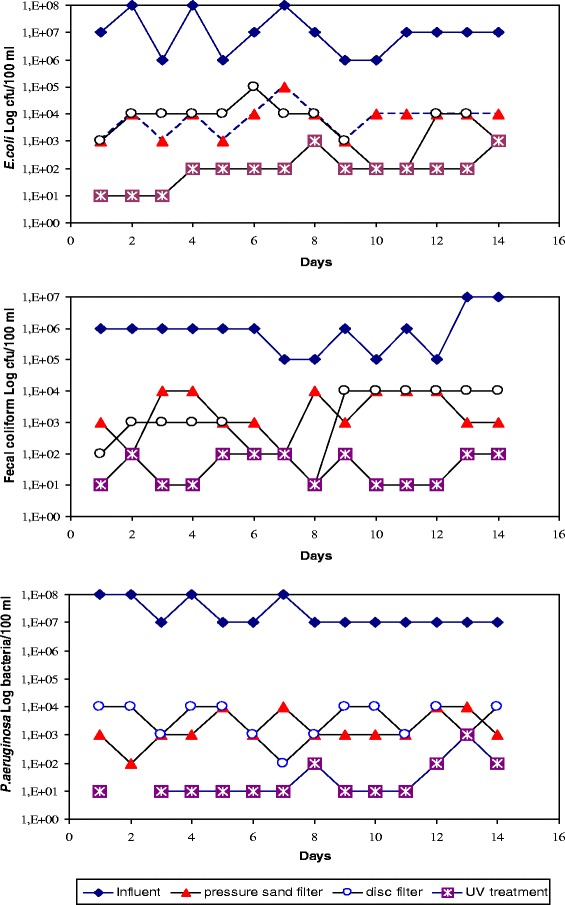
***E. coli,*** **feacal coliform counts (Log cfu/100 ml) and** ***P. aeruginosa*** **counts (Log bacteria/100 ml) in influent from disc filter, pressure sand filter and UV treatment.** y: Reduction = *N/N*_*0*_ with *N*; Number of micro-organisms at the instant *T*; *N*_*0*_; Number of micro-organisms at the instant *T* = 0.

All the bacterial counts were carried out for influent and effluent delivered by the two filtration systems under study. Figure 6 compared the result of the bacteriological concentrations (*E. coli,* feacal coliform and *P. aeruginosa*) in the influent and effluent of the disc filter and of the pressure sand filter, respectively, and indicated a substantial bacterial count reduction between the entrance and the exit of the two-macrofiltration systems. Therefore, these observed results demonstrated that both macrofiltration systems showed a high removal capacity that exceeds in some cases 4 U-Log for *E. coli and P. aeruginosa;* but for feacal coliform, their inactivation rate is around 3 U-Log.

As stated in Figure 6, no important differences were found between counts in the effluent from disc filter and pressure sand filter for *E. coli,* feacal coliform and *P. aeruginosa*. Similarly, no significant differences were found between removal percentages of the two-macrofiltration systems. Despite this apparently good typical removal of 4 U-Log, it appeared on average for a N_o_ of 10^7^ CFU/ml in the case of *E. coli* for example, the remaining number of 10^3^ bacteria/ml could cause serious health and environmental problems.

### Bacteriological validation of UV reactor

Because the UV dose delivered by the UV irradiation system is a complex function of many variables, the UV reactor validation was used to demonstrate the disinfection efficacy and performance. In addition, the disinfection performance of a UV system is determined based on a series of bacteriological challenges that are conducted on a pilot-scale UV reactor. During a validation, a UV system is evaluated under various operational conditions that may include flow rate, **UVT**, lamp power (or relative lamp output), water level, and number of operational lamps. During each test condition, water samples were collected at the entrance and the exit of the UV reactor to quantify the rate of inactivation of a target challenge bacteria associated to the specific operational conditions.

In this study and as shown in Figure 2, the UV bacterial inactivation rates for *E. coli*, feacal coliforms and *P. aeruginosa* (log reduction) obtained, respectively, at the exit of the disc filter or pressure sand filter and at different dose UV, did not noticed any meaningful differences. It seems useful to adopt the secondary effluent at the exit of the pressure sand filter as a basic sample for the bacteriological tests adopted for the three selected bacteria studied, and for future daily monitoring of the UV reactor.

On the other hand, several standardized international guidelines stipulate that the reuse of wastewater requires a decrease in the number of indicator bacteria of about 3 U-log. However, the complexity of the present processes and requirements for environmental safety, microbiology, public health and even industry, need the introduction of advanced monitoring systems, based on monitoring methodologies built on the principle of analytical redundancy. For this reason, a second standard requires a reduction ratio of the number of *P. aeruginosa* of the order of 4 U-log for treated wastewater reuse [8].

As stated in Figures 4 and 5, an almost total elimination of these indicators would be achieved with UV doses over than 96 mJ/cm^2^, corresponding to a UV exposure of 16 min. Therefore, the use of a dose of 96 mJ/cm^2^ is necessary, firstly to calculate the performance of the UV reactor, and secondly to obtain water free from pathogen bacteria and meets the standards recommended by several standardized international guidelines.

In this sense, the daily bacteriological monitoring of the performance of the UV reactor by the use of a dose of 96 mJ/cm^2^, corresponding to an exposure of 16 min and considering the wastewater at the outlet of pressure sand filter, was finalized and shown in Figure 6. It appeared from the Figure 6 that the rate of inactivation, on average achieved, were around 3 U-Log for *E. coli,* feacal coliforms and *P. aeruginosa*, respectively. Therefore, the average concentration remaining in the treated wastewater at the exit of the UV reactor is less than a 1000 cfu/100 ml of *E. coli and* feacal coliform. For *P. aeruginosa*, this rate left is less than 100 bacteria/100 ml. These values agree with the range recommended by several standardized international guidelines [8].

Also from the Figure 6, it seemed obvious that *E. coli* and feacal coliforms were signaled as the UV most resistant bacteria; contrariwise, *P. aeruginosa* was found to be the most sensitive. In dissimilarity, authors [15] and [16] reported that *P. aeruginosa* is very resistant to UV irradiation, which contradicts the above observations that *P. aeruginosa* is more sensitive to UV irradiation than *E. coli* and feacal coliform. This discrepancy in the results could be explained by the fact that *E. coli* and feacal coliform forms aggregates in the treated effluent, while *P. aeruginosa* exists either as discrete cells or as cell pairs. This self-aggregation ability provides an advantage to the *E. coli* and feacal coliform over *P. aeruginosa,* since the concentration of suspended matter and the state of aggregation could shelter and protect the bacteria from UV radiation in the effluent [17].

## Conclusions

Results for the three categories of bacteria studied lead to similar conclusions. The macrofiltration systems (a disc filter or sand pressure filter) allowed a good removal of feacal bacteria (*E. coli,* feacal coliforms) and of *P. aeruginosa*, although the bacterial counts indicated the continued appearance and presence of these bacteria in the treated effluent. This result pleads and advocates the need for a disinfection treatment.

On the other side, the quality of wastewater acquired from a disc filter or sand pressure was dependent on the initial quality of the effluent to be treated. Consequently, bacteriological quality of water treated by macrofiltration will vary depending on the type of effluent used. It was consequently not possible to guarantee a specific quality of the effluents issued from the treatment of macrofiltration, and this fact affected some parameters such as turbidity and suspended solids [18]. Moreover, variability in the quality of macrofiltration effluent might affect seriously the performance of disinfection technologies, for instance UV radiation, which required a specific quality of effluent to be treated, particularly with regard to parameters such as turbidity, UV transmittance and suspended solids content [3,18].

A further consideration is that the UV radiation dose to be applied was affected by high variations in the number of microbes needful for elimination, as observed in the effluents under study, and this fact renders the treatment yet more problematic. This type of drawback may be avoided by employing an optimized good UV reactor as disinfection systems.

The use of a UV dose of 96 mJ/cm^2^, corresponding to an exposure of 16 min of the secondary effluent at the exit of pressure sand filter, released water of a very good bacteriological quality and meets the standards recommended by several standardized international guidelines.

The greatest obstacle to these technologies remains to be the economic cost of both installations and system operation. In recent years, however, costs have fallen considerably due to technological improvements, particularly with regard to the macrofiltration systems and modular design. Macrofiltration systems followed by a UV reactor may now be seen as a practicable option for a large number of applications inside in the field of operations of wastewater reutilization [19].
